# Cancer Stem Cells in Moderately Differentiated Lip Squamous Cell Carcinoma Express Components of the Renin–Angiotensin System

**DOI:** 10.3389/fsurg.2017.00030

**Published:** 2017-06-06

**Authors:** Rachna S. Ram, Helen D. Brasch, Jonathan C. Dunne, Paul F. Davis, Swee T. Tan, Tinte Itinteang

**Affiliations:** ^1^Gillies McIndoe Research Institute, Wellington, New Zealand; ^2^Wellington Regional Plastic, Maxillofacial and Burns Unit, Hutt Hospital, Wellington, New Zealand

**Keywords:** lip, squamous cell carcinoma, renin–angiotensin system, cancer stem cells, oral cavity, cancer

## Abstract

**Aim:**

We investigated the expression of the renin–angiotensin system (RAS) by cancer stem cell (CSC) subpopulations we have identified in moderately differentiated lip squamous cell carcinoma (MDLSCC).

**Method:**

Ten MDLSCC samples underwent 3,3-diaminobenzidine (DAB) and immunofluorescent immunohistochemical (IHC) staining for (pro)renin receptor (PRR), angiotensin-converting enzyme (ACE), angiotensin II receptor 1 (ATIIR1), and receptor 2 (ATIIR2). NanoString analysis and Western blotting (WB) were performed on six MDLSCC samples for gene and protein expression, respectively.

**Results:**

IHC staining showed expression of PRR, ATIIR1, and ATIIR2 on cells within the tumor nests (TNs) and the stroma. ACE was localized to the microvessels within the stroma. WB detected PRR, ACE, and ATIIR2. NanoString analysis confirmed gene expression of PRR, ACE, and ATIIR1.

**Conclusion:**

Components of the RAS: PRR, ATIIR1, and ATIIR2 are expressed on two CSC subpopulations in MDLSCC, one within the TNs and the other within the stroma. The endothelium of the microvessels within the stroma expresses ACE.

## Introduction

Oral cavity cancer is the sixth most common cancer globally with the majority being squamous cell carcinoma (SCC) (1, 2). Oral cavity SCC (OCSCC) affects the lip, oral tongue, floor of mouth, hard palate, buccal mucosa, maxillary and mandibular alveolus, and the retromolar trigone (1, 3). The incidence of lip cancer is highest among white populations in Canada and Australia (2).

Risk and etiological factors for OCSCC include allelic imbalance involving tumor suppressor genes and oncogenes (4), carcinogen metabolizing enzymes (5), tobacco (6), immune deficiency (7), UV exposure (7), and human papilloma virus infection (8, 9). Treatment for lip SCC includes surgery and radiotherapy singly as primary treatment, or in combination for advanced or higher grade lesions (10). While 5-year survival rates for oral cavity cancer are around 50–60% (1, 3), lip cancers have 5-year survival rates of up to 90% (2).

The cancer stem cell (CSC) concept proposes that a population of cells within a tumor are tumorigenic (11, 12). CSCs have been proposed as the origin of cancer cells and account for tumor resistance to therapeutic agents and give rise to metastases (11, 13). Although there are no distinctive markers that differentiate normal stem cells from CSCs, several stem cell markers have been used to identify CSCs in OCSCC (14, 15).

We have recently demonstrated the presence of three CSC subpopulations within moderately differentiated lip SCC (MDLSCC): a CD44^+^/SALL4^+^/NANOG^+^/pSTAT3^+^/SOX2^+^/OCT4^−^ CSC sub-population within the tumor nests (TNs), a CD44^+^/SALL4^+^/NANOG^+^/pSTAT3^+^/SOX2^+^/OCT4^−^ CSC subpopulation, and a CD44^+^/SALL4^+^/NANOG^+^/pSTAT3^+^/SOX2^+^/OCT4^+^ CSC subpopulation within the stroma, between the TNs (16). These findings are in keeping with our previous characterization of CSCs in oral tongue (17, 18) SCC.

The renin–angiotensin system (RAS) is a major hormonal system that regulates blood pressure and fluid balance through its effects in the kidney, cardiovascular, and central nervous systems (19, 20). Angiotensinogen is cleaved by renin to form angiotensin I (ATI) via the (pro)renin receptor (PRR) (19). Angiotensin-converting enzyme (ACE) then cleaves ATI to angiotensin II (ATII). ATII is the main effector of the RAS and mediates its effects through angiotensin II receptor 1 (ATIIR1) and angiotensin II receptor 2 (ATIIR2) (19, 20).

Expression of several components of the RAS have been demonstrated in abnormal tissues such as mammary hyperplasia and ductal carcinoma *in situ* (21) and cancer cells in human breast, lung, pancreas, prostate, skin cancer, and glioblastoma multiforme (GBM) (21–23). These components have also been correlated with inflammation, tumor growth, and angiogenesis (21, 24). Although the RAS has been implicated in carcinogenesis (20, 25), there are little data demonstrating the presence of the RAS in lip SCC.

This study investigated the expression of components of the RAS: PRR, ACE, ATIIR1, ATIIR2, in the CSC subpopulations within MDLSCC that we had previously characterized (16).

## Materials and Methods

### Tissue Samples

Moderately differentiated lip squamous cell carcinoma samples from one female and nine male patients aged 46–94 (mean, 64.4) years sourced from the Gillies McIndoe Research Institute Tissue Bank, were used for this study, which was approved by the Central Regional Health and Disability Ethics Committee (ref. no. 12/CEN/74).

### Histochemical and Immunohistochemical (IHC) Staining

Hematoxylin and eosin (H&E) staining was performed on 4-μm thick formalin-fixed paraffin-embedded sections of MDLSCC samples by an anatomical pathologist (HDB) to confirm the appropriate histological grade and to identify areas of SCC within the tissue sections. 3,3-Diaminobenzidine (DAB) IHC staining was performed on the sections using primary antibodies for PRR (1:100; cat # HPA003156, Sigma, St. Louis, MO, USA), ACE (1:100; cat# MCA2054, AbD Serotec, Raleigh, NC, USA), ATIIR1 (1:30; cat# ab9391, Abcam), ATIIR2 (1:2,000; cat# NBP1-77368, Novus Biologicals, Littleton, CO, USA), and ETS-related gene (ERG; 1:200; cat# EP111, Cell Marque, Rocklin, CA, USA). All slides were mounted in Surgipath Micromount (cat# 3801732, Leica, Richmond, IL, USA).

To determine co-expression of two proteins, immunofluorescent (IF) IHC staining was performed on two MDLSCC samples from the original cohort of 10 patients used for DAB IHC staining. This was done utilizing a combination of VectaFluor Excel anti-mouse 488 (ready-to-use; cat# VEDK2488, Vector Laboratories, Burlingame, CA, USA) and Alexa Fluor anti-rabbit 594 (1:500; cat# A21207, Life Technologies, Carlsbad, CA, USA) to detect combinations that included OCT4 (1:1,000; cat# EPR2054, ab109183, Abcam), SALL4 (1:30; cat# CM385M-16, Cell Marque, Rocklin, CA, USA), SOX2 (1:200; cat# PA1-094, Thermo Fisher Scientific, Waltham, MA, USA), and ERG (1:200; cat# EP111, Cell Marque) with PRR (1:2,000; cat#, ab40790, Abcam), ACE (1:100; cat# MCA2054, AbD Serotec), ATIIR1 (1:30; cat# ab9391, Abcam), and ATIIR2 (1:2,000; cat# NBPI 1-12677368, Novus Biologicals). All IF IHC-stained slides were mounted in Vectashield HardSet mounting medium with 4′,6-diamidino-2-phenylindole (Vector Laboratories).

Positive human control tissues used for primary antibodies for IHC staining were placenta for PRR and ERG; liver for ATIIR1, kidney for ACE and ATIIR2, skin for SOX2, and seminoma for OCT4 and SALL4. A negative control by omitting the primary antibody on a MDLSCC sample was also prepared (data not shown).

All antibodies were diluted with Bond primary antibody diluent (cat# AR9352, 143 Leica) and all DAB and IF IHC staining was carried out on the Leica Bond Rx autostainer as previously described (26).

### Western Blotting

Total protein was extracted from six MDLSCC specimens, from the original cohort of 10 patients used for DAB IHC staining, by homogenization in ice-cold RIPA buffer (Sigma-Aldrich, St. Louis, MA, USA) containing 10 mM dithiothreitol (Sigma-Aldrich) and 1× HALT protease and phosphatase inhibitor cocktail (Thermo Fisher Scientific). Western blotting (WB) analysis was performed as described previously (27), with changes to the antibodies used as follows: PRR (1:500; cat# HPA003156, Sigma-Aldrich), ACE (1:200; cat# sc-12184, Santa Cruz Biotechnology, Dallas, TX, USA), ATIIR1 (1:200; cat# sc-1173, Santa Cruz Biotechnology), ATIIR2 (1:5,000; cat# ab92445, Abcam), and β-actin (1:2,000; cat# ab8226, Abcam). Appropriate secondary antibodies used were goat anti-rabbit horseradish peroxidase (HRP) conjugate (1:10,000; cat# A16110, Life Technologies), donkey anti-goat HRP conjugate (1:5,000; cat# ab97120, Abcam) or Alexa Fluor^®^ 647 rabbit anti-mouse (1:2,000; cat# A21239, Life Technologies). All primary and secondary antibodies were diluted in TBS containing 0.1% Tween-20. Detection of the HRP-conjugated secondary antibodies was achieved using Clarity™ Western ECL substrate (Bio-Rad, Hercules, CA, USA). Membranes were imaged using the ChemiDoc MP imaging system (Bio-Rad).

### Nanostring Gene Analysis

Six snap-frozen MDLSCC samples from the original cohort of 10 patients used for DAB IHC staining were utilized for isolation of total RNA for NanoString nCounter™ Gene Expression Assay (NanoString Technologies, Seattle, WA, USA). RNA was extracted from tissues using a RNeasy Mini Kit (Qiagen, Hilden, Germany) and quantitated by Qubit^®^ 2.0 Fluorometer (Life Technologies). This RNA was subjected to NanoString nCounter™ gene expression assay completed by New Zealand Genomics Ltd. (Dunedin, NZ), according to the manufacturer’s protocol. Probes for *PRR* (ATP6AP2, NM_005765.2), *ACE* (NM_000789.2), *ATIIR1* (AGTR1, NM_000685.3), *ATIIR2* (AGTR2, NM_000686.3), and the housekeeping gene *GUSB* (NM_000181.1) were designed and synthesized by NanoString Technologies. Data analysis was done using nSolver™ software (NanoString Technologies) with standard settings. Results were normalized against the housekeeping gene and graphed using Excel (Microsoft Office 2013).

### Image Analysis

The IHC-stained slides were viewed and imaged using an Olympus BX53 light microscope (Olympus, Tokyo, Japan) and an Olympus FV1200 confocal laser-scanning microscope (Olympus) was used for IF IHC-stained slides. The IF IHC-stained images were processed with CellSens Dimension 1.11 software using the 2D deconvolution algorithm (Olympus).

## Results

### IHC Staining

**Figure 1:**
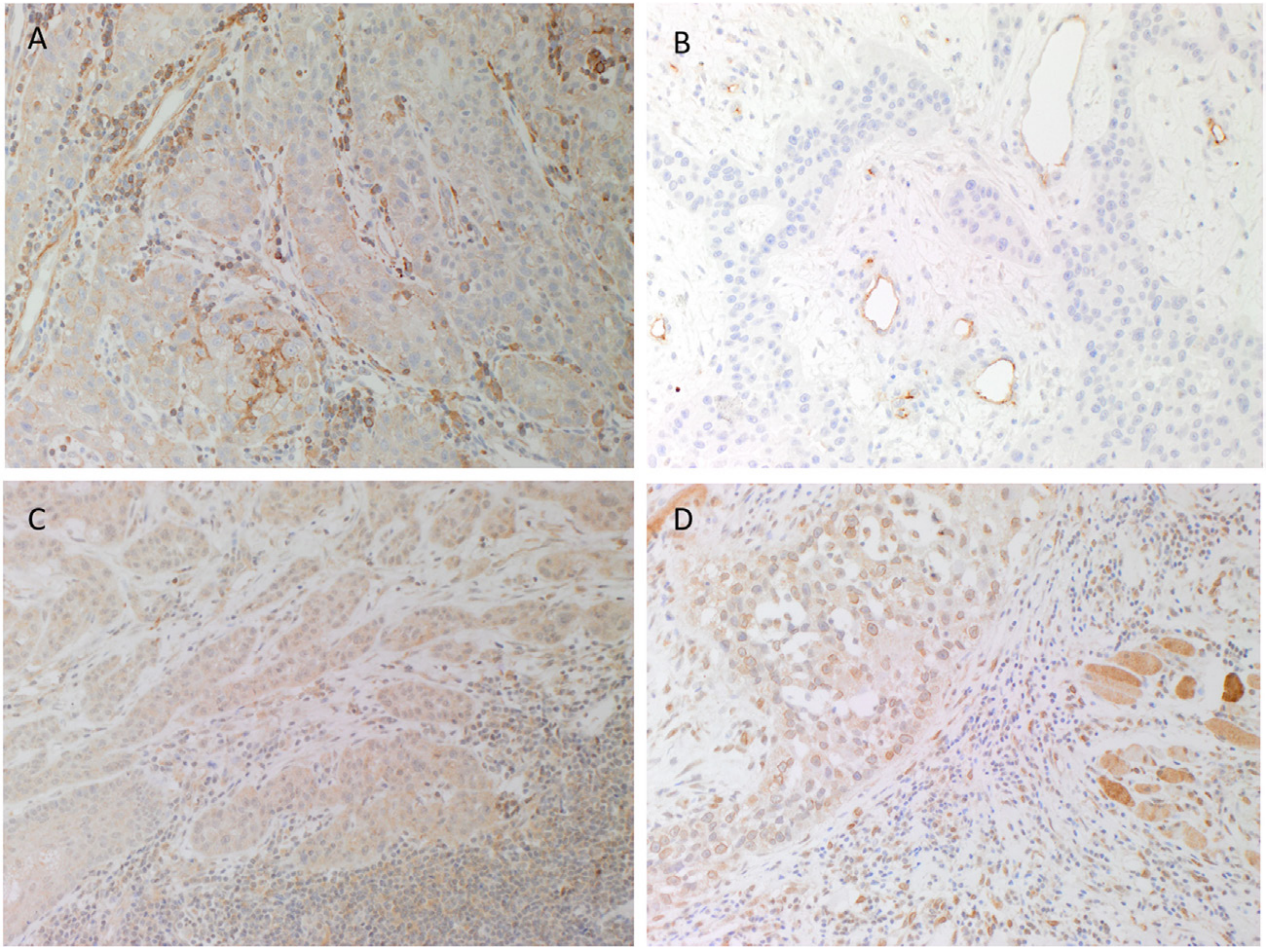
**Representative 3,3-diaminobenzidine immunohistochemical-stained sections of moderately differentiated lip cancer samples showing cytoplasmic expression of (pro)renin receptor [(A), brown] within the tumor nests (TNs) and cells within the stroma**. Angiotensin-converting enzyme [**(B)**, brown] was expressed on the endothelium of the microvessels within the stroma but not the TNs. Angiotensin II receptor 1 [**(C)**, brown] showed weak to moderate cytoplasmic and nuclear staining by cells within the TNs and cells within the stroma. Strong perinuclear and cytoplasmic staining for angiotensin II receptor 2 [**(D)**, brown] was present in cells within the TNs and the stroma, and skeletal tissue. Nuclei were counterstained with hematoxylin [**(A–D)**, blue]. Original magnification: 200×.

H&E staining confirmed the histological grade and the presence of MDLSCC for all 10 samples. DAB IHC staining showed weak to moderate cytoplasmic expression of PRR localized predominantly to cells within the TNs and occasional cells within the stroma (Figure 1A, brown). ACE was present within the endothelium of the microvessels within the stroma between the TNs (Figure 1B, brown) but not the cells within the TNs. Weak to moderate cytoplasmic and nuclear staining of ATIIR1 was observed in cells within the TNs and cells within the stroma (Figure 1C, brown). There was strong perinuclear and cytoplasmic staining of ATIIR2 in cells within the TNs, the stroma and skeletal muscle (Figure 1D, brown). A summary of staining patterns across all 10 samples is given in Table S1 in Supplementary Material.

Positive human control tissues demonstrated the expected staining pattern in placenta for PRR (Figure S1A in Supplementary Material) and ERG (data not shown), liver for ACE (Figure S1B in Supplementary Material) and ATIIR1 (Figure S1C in Supplementary Material, brown) and kidney for ATIIR2 (Figure S1D in Supplementary Material, brown). The omission of the primary antibody in a MDLSCC sample provided a control for the secondary antibody (data not shown).

### IF IHC Staining

**Figure 2:**
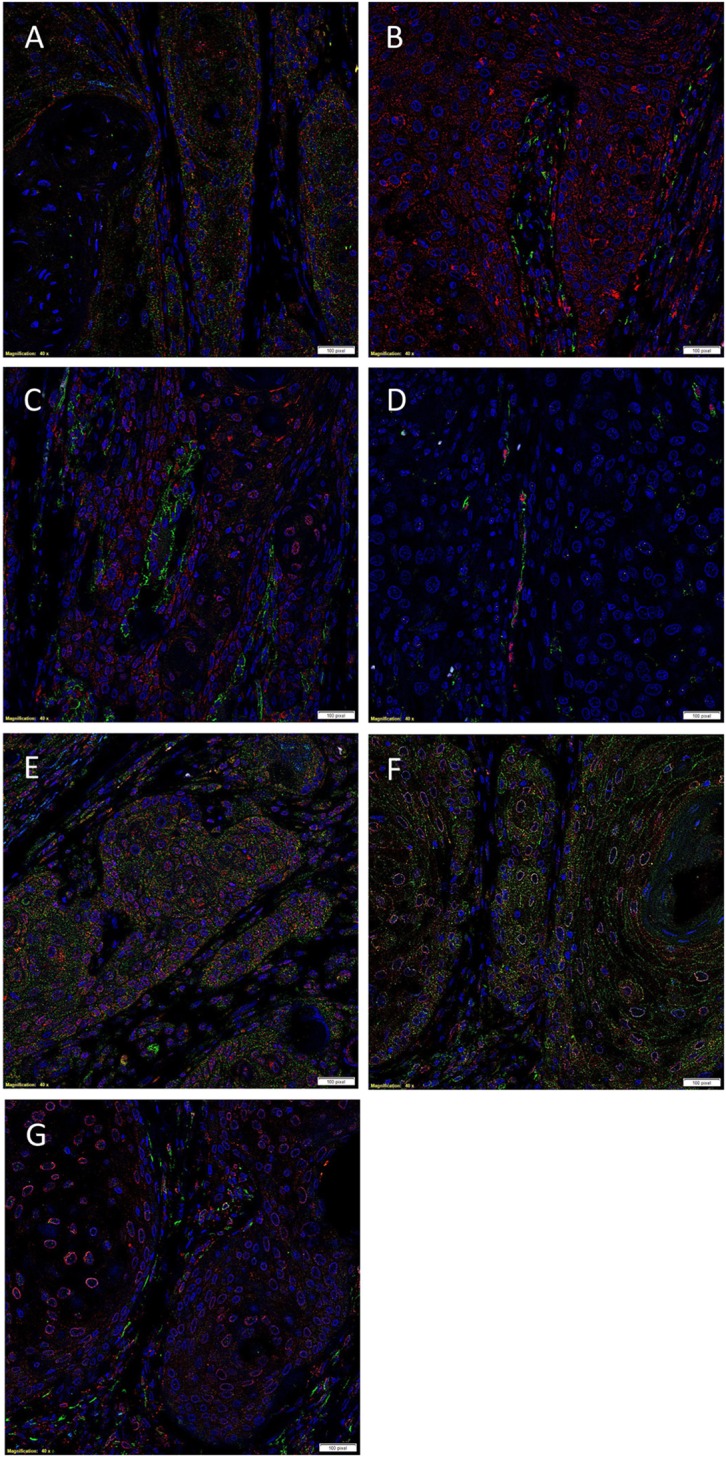
**Representative immunofluorescent immunohistochemical-stained sections of moderately differentiated lip squamous cell carcinoma demonstrating expression of (pro)renin receptor (PRR) [(A,B), red] by cells within the tumor nests (TNs) and the cells within the stroma that expressed SALL4 [(A), green]**. PRR was also expressed by cells within the stroma that expressed OCT4 [**(B)**, green]. Angiotensin-converting enzyme (ACE) [**(C)**, green] was expressed on the endothelium of the microvessels which expressed SOX2 [**(C)**, red], within the stroma. Staining with ETS-related gene [**(G)**, red] confirmed that ACE [**(G)**, green] was expressed by the endothelium of the microvessels within the stroma. Angiotensin II receptor 1 [**(D)**, green] was demonstrated in cells within the TNs and occasional microvessels that stained positively for SOX2 [**(D)**, red]. Angiotensin II receptor 2 [**(E,F)**, red] was expressed by cells within the TNs, and cells within the stroma that expressed SALL4 [**(E)**, green] and those that expressed OCT4 [**(F)**, green]. Cell nuclei were counterstained with 4′,6′-diamidino-2-phenylindole [**(A**–**G)**, blue]. Scale bars: 20 µm.

Double IF IHC staining showed that PRR (Figure 2A, red) was expressed by the SALL4^+^ cells (Figure 2A, green) within the TNs and the stroma. PRR (Figure 2B, red) was also detected in the OCT4^+^ CSC subpopulation (Figure 2B, green) and a separate OCT^−^ CSC subpopulation (Figure 2B, red) within the stroma. Expression of ACE (Figure 2C, green) was observed in endothelium of the microvessels that also expressed SOX2 (Figure 2C, red). Expression of ACE was localized exclusively to the endothelium of the microvessels that expressed the endothelial marker ERG (Figure 2D, red). ATIIR1 (Figure 2E, green) was expressed by cells within the TNs as well as the microvessels that also expressed SOX2 (Figure 2E, red). Perinuclear expression of ATIIR2 (Figures 2F,G, red) was demonstrated in cells within the TNs that expressed SALL4 (Figure 2F, green) as well as cells within the stroma, regardless of whether they were OCT4^+^ (Figure 2G, green) or OCT4^−^ (Figure 2G, red). Separated IF IHC images of Figure 2 are shown in Figure S2 in Supplementary Material.

### Western Blotting

**Figure 3:**
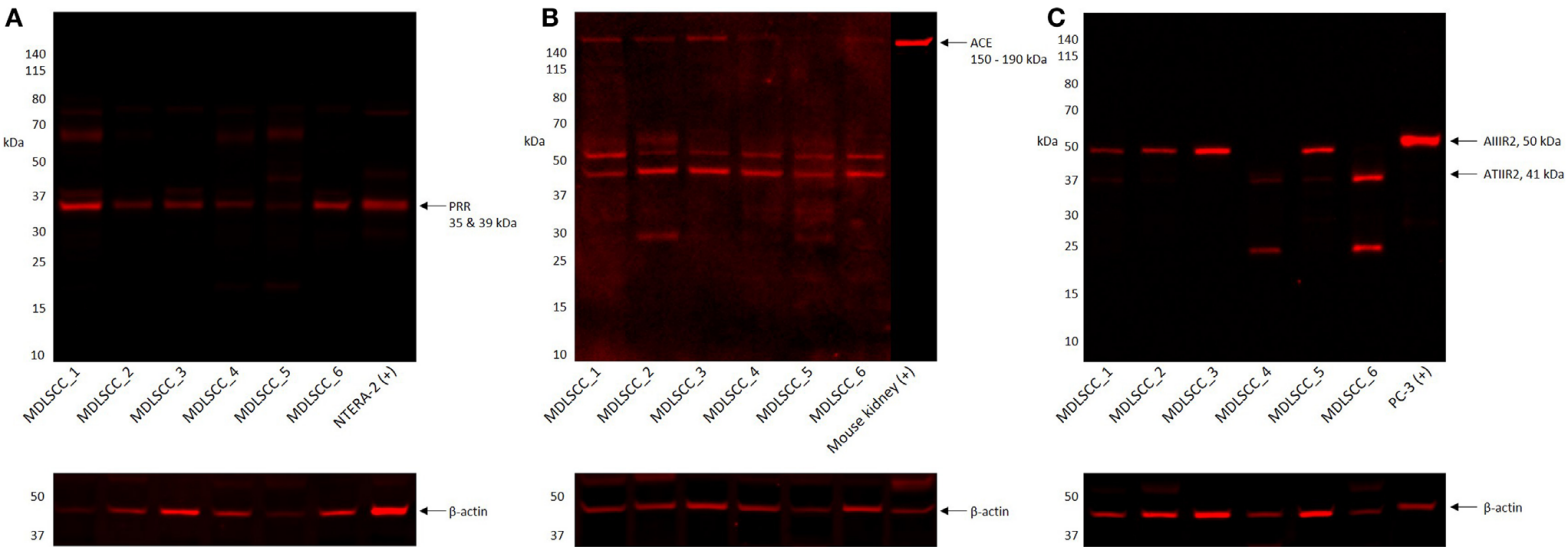
**Western blot images of moderately differentiated lip squamous cell carcinoma total protein extracts, probed for (pro)renin receptor (PRR) (A), angiotensin-converting enzyme (ACE) (B), and angiotensin II receptor 2 (ATIIR2) (C), detected with horseradish peroxidase-conjugated goat anti-rabbit (A,B) or donkey anti-491 goat (C) secondary antibody**. β-actin was used as the loading control and detected using Alexa Fluor^®^ 647 rabbit anti-mouse secondary antibody.

Western blotting confirmed the presence of PRR at an expected molecular weight of 35 kDa (Figure 3A) within the extracts of all six MDLSCC samples examined. ACE (Figure 3B) was detected in five of the six MDLSCC samples analyzed, at the expected molecular weight of 195 kDa. ATIIRI was detected at 41 kDa in five samples, however, its proximity to the control ruled out significance (data not shown). ATIIR2 was detected in four of the six MDLSCC samples at approximately 50 kDa (Figure 3C), which is larger than the native form of the protein and may be due to glycosylation. β-actin confirmed approximately equal total protein loading in all samples (Figures 3A–C).

### Nanostring Gene Analysis

**Figure 4:**
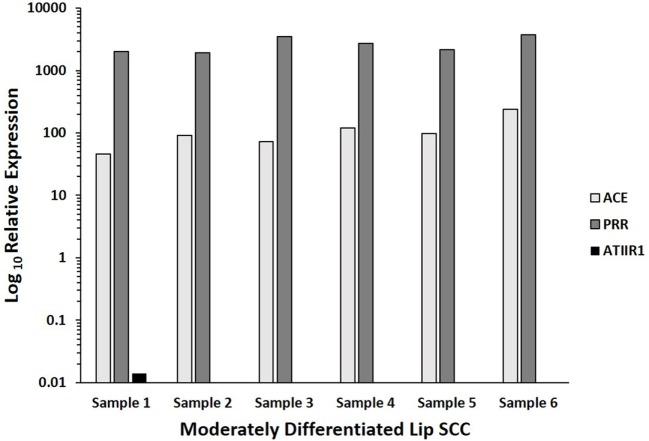
**Log_10_ relative expression of mRNA transcripts of components of the renin–angiotensin system in six moderately differentiated lip squamous cell carcinoma samples, over the CLTC housekeeper**. Angiotensin II receptor 1 (ATIIR1) was detected in one sample. Transcriptional profiling confirmed the presence of (pro)renin receptor (PRR) and angiotensin-converting enzyme (ACE) mRNA in all six samples, and ATIIR1 in one sample. Angiotensin II receptor 2 mRNA was below detectable levels within all six samples.

NanoString gene analysis for *PRR, ACE, ATIIR1*, and *ATIIR2* performed on six MDLSCC samples from the original cohort of 10 patients used for DAB IHC staining, confirmed the presence of *PRR* and *ACE* mRNA in all six samples, and *ATIIR1* mRNA in one sample. *ATIIR2* mRNA was below detectable levels in all six samples (Figure 4).

## Discussion

The RAS is a complex endocrine system that is a therapeutic target for hypertension and has been associated with the metabolic system (28), inflammation (29, 30), and carcinogenesis (22, 25). Although physiologically the liver is the primary source of circulating AGN, it is also expressed by other cell types including adipocytes, mesangial cells, and proximal tubule epithelial cells of the kidney, and neurons and glial cells in the brain (28).

Components of the RAS were identified by IHC staining in all 10 MDLSCC samples examined in our study. Interestingly, ACE was localized to the endothelium of the microvessels located within the stroma, between the TNs. The presence of ACE on the endothelium of the microvessels within the stroma that also expresses the ESC marker SOX2 is intriguing. This is consistent with recent reports of a primitive endothelial phenotype in close proximity to colorectal tumors (31) suggesting its role in regulating epithelial to mesenchymal transition by influencing angiogenesis (20, 22). However, this remains the topic of further investigation.

Expression of PRR was seen in the SALL4^+^/OCT4^−^ CSCs within the TNs and the SALL4^+^/OCT4^+^ CSCs within the stroma. Expression of ATIIR1 was present in SOX2^+^ CSCs within the TNs and the endothelium of the microvessesls within the stroma. ATIIR2 was expressed by the SALL4^+^/OCT4^−^ CSCs within the TNs, as well as the SALL4^+^/OCT4^+^ CSCs within the stroma. The expression of PRR, ACE, and ATIIR2 was supported by WB. While the presence of mRNA for PRR and ACE was confirmed in all six samples, ATIIR1 was detected by NanoString gene analysis in only one sample and ATIIR2 was below detectable levels in all samples. This could be attributed to degradation of mRNA or lower rate of mRNA transcription compared to protein translation. Although this study demonstrates novel findings of the expression of components of the RAS by the CSC subpopulations in MDLSCC, its descriptive nature necessitates further work into the functional role of the RAS, including a larger sample size.

The hallmarks of cancer (12, 32) are the distinct capabilities of cells to proliferate, survive, and disseminate, and the regulation of these features by the RAS suggests its direct effect on angiogenesis (25, 33).

Recent studies have demonstrated the presence of components of RAS in breast cancer (22, 34). To the best of our knowledge, this is the first report demonstrating the expression of components of the RAS by the CSC subpopulations within MDLSCC. The localization of the RAS to the CSC subpopulations within MDLSCC adds to the similar finding in oral tongue (35) and buccal mucosal (27) SCC and GBM (36). This would suggest that CSCs may be a novel therapeutic target for this cancer by using drugs that modulate the RAS.

Previous studies have demonstrated an association of the use of ACE inhibitors and angiotensin II receptor 1 blockers with reduced risk of development of skin SCC (37), and β-blockers administration which reduce renin levels (38), is associated with improved survival of patients with malignant melanoma (39). Although there is currently no literature on the potential role RAS modulators on lip SCC, propranolol has been shown to reduce viability, induce apoptosis, and inhibit production of the proangiogenic protein VEGF in head and neck SCC cell lines (40). Further work is needed to determine the validity of targeting CSCs by modulating the RAS in MDLSCC and other types of cancer as a novel therapeutic approach.

## Ethics Statement

This study was approved by the Central Regional Health and Disability Ethics Committee.

## Author Contributions

TI and ST formulated the study hypothesis and designed the study. RR, HB, TI, and ST interpreted the DAB and IF IHC data. JD performed WB analysis. JD, TI, PD, and ST interpreted the WB data. TI and ST interpreted the NanoString data. RR, TI, and ST drafted the manuscript. All the authors commented on and approved the manuscript.

## Conflict of Interest Statement

The authors declare that the research was conducted in the absence of any commercial or financial relationships that could be construed as a potential conflict of interest. TI, PD, and ST are inventors of the PCT patent application (No. PCT/NZ2015/050108) Cancer Diagnosis and Therapy.

## Supplementary Material

Figure S1 **Representative 3,3-diaminobenzidine immunohistochemical-stained sections of positive control human tissues showing positive staining in placenta for PRR (A), kidney for ACE (B, brown) and ATIIR2 (D, brown), and liver for ATIIR1 (C, brown)**.

Figure S2 **Split images of immunofluorescent immunohistochemical-stained sections moderately differentiated lip squamous cell carcinoma demonstrating the expression of for PRR (A,C, red), SALL4 (B,L, green), OCT4 (D,H,N, green), SOX2 (E,I, red), ACE (F, green), ERG (G, red), ATIIR1 (J, green), ATIIR2 (K,M, red) shown in Figure 2**. Cell nuclei were counterstained with 4′,6′-diamidino-2-phenylindole (A–N, blue). Scale bars: 20 μm.
