# Supplementary material for: Cancer Stem Cells in Moderately Differentiated Lip Squamous Cell Carcinoma Express Components of the Renin–Angiotensin System
Source: Front Surg. 2017 Jun 6;4:30. doi: 10.3389/fsurg.2017.00030 (PMC5459876; doi:10.3389/fsurg.2017.00030)
Supplement: Supplementary file 1 [file table_1.docx]

**Supplementary Table 1** 3,3-Diaminobenzidine Immunohistochemical Staining Patterns of Components of the Renin-Angiotensin System in 10 Patients

| **Patients** | **Components of the Renin-Angiotensin System** | | | |
| --- | --- | --- | --- | --- |
|  | **ACE** | **PRR** | **ATIIR1** | **ATIIR2** |
| 1 | ++ microvessels | ++ cytoplasmic in cells in TNs | + cytoplasmic in cells in TNs  + microvessels | + cytoplasmic and perinuclear in cells in TNs  + microvessels |
| 2 | ++ microvessels | + cytoplasmic in cells in TNs  + focal in cells in stroma | ++ cytoplasmic and nuclear in cells in TNs  ++ diffuse in cells in stroma  ++ microvessels | + cytoplasmic and perinuclear in cells in TNs  + focal in cells in stroma  + microvessels |
| 3 | ++ microvessels | + cytoplasmic in cells in TNs | + cytoplasmic in cells in TNs  + diffuse in cells in stroma  + microvessels | + cytoplasmic and perinuclear in cells in TNs  ++ diffuse in cells in stroma  + microvessels |
| 4 | ++ microvessels | ++ cytoplasmic in cells in TNs | ++ cytoplasm in cells in TNs  ++ Diffuse in cells in stroma  ++ microvessels | + perinuclear in cells in TNs  ++ focal in cells in stroma  ++ microvessels |
| 5 | ++ microvessels | + cytoplasmic in cells in TNs  + focal in cells in stroma | + cytoplasmic and nuclear in cells in TNs  ++ diffuse in cells in stroma  ++ microvessels | + cytoplasmic and perinuclear in cells in TNs  ++ diffuse in cells in stroma  + microvessels |
| 6 | ++ microvessels | ++ cytoplasmic in cells in TNs  + focal in cells in stroma | + cytoplasmic in cells in TNs  ++ diffuse in cells in stroma  ++ microvessels | + perinuclear in cells in TNs  ++ diffuse in cells in stroma  + microvessels |
| 7 | ++ microvessels | ++ cytoplasmic in cells in TNs  ++ focal in cells in stroma | + cytoplasmic in cells in TNs  ++ diffuse in cells in stroma  + microvessels | + cytoplasmic and perinuclear in cells in TNs  ++ focal in cells in stroma  + microvessels |
| 8 | ++ microvessels | + cytoplasmic in cells in TNs  + focal in cells in stroma | + cytoplasmic in cells in TNs  + diffuse in cells in stroma  + microvessels | + cytoplasmic in cells in TNs  - in cells in stroma  + microvessels |
| 9 | ++ microvessels | ++ cytoplasmic and perinuclear in cells in TNs | + cytoplasmic in cells in TNs  ++ diffuse in cells in stroma  ++ microvessels | ++ cytoplasmic and perinuclear in cells in TNs  ++ diffuse in cells in stroma  ++ microvessels |
| 10 | ++ microvessels | ++ T cytoplasmic in cells in TNs  ++ focal in cells in stroma | - in cells in TNs  + focal in cells in stroma  + microvessels | + cytoplasmic and perinuclear in cells in TNs  ++ diffuse in cells in stroma  + microvessels |

TNs, tumor nests; ACE, angiotensin converting enzyme; PRR, pro(renin) receptor; ATIIR1, angiotensin II receptor 1; ATIIR2, angiotensin II receptor 2
