# Supplementary figures and images for: Cancer Stem Cells in Moderately Differentiated Lip Squamous Cell Carcinoma Express Components of the Renin–Angiotensin System
Source: Front Surg. 2017 Jun 6;4:30. doi: 10.3389/fsurg.2017.00030 (PMC5459876; doi:10.3389/fsurg.2017.00030)

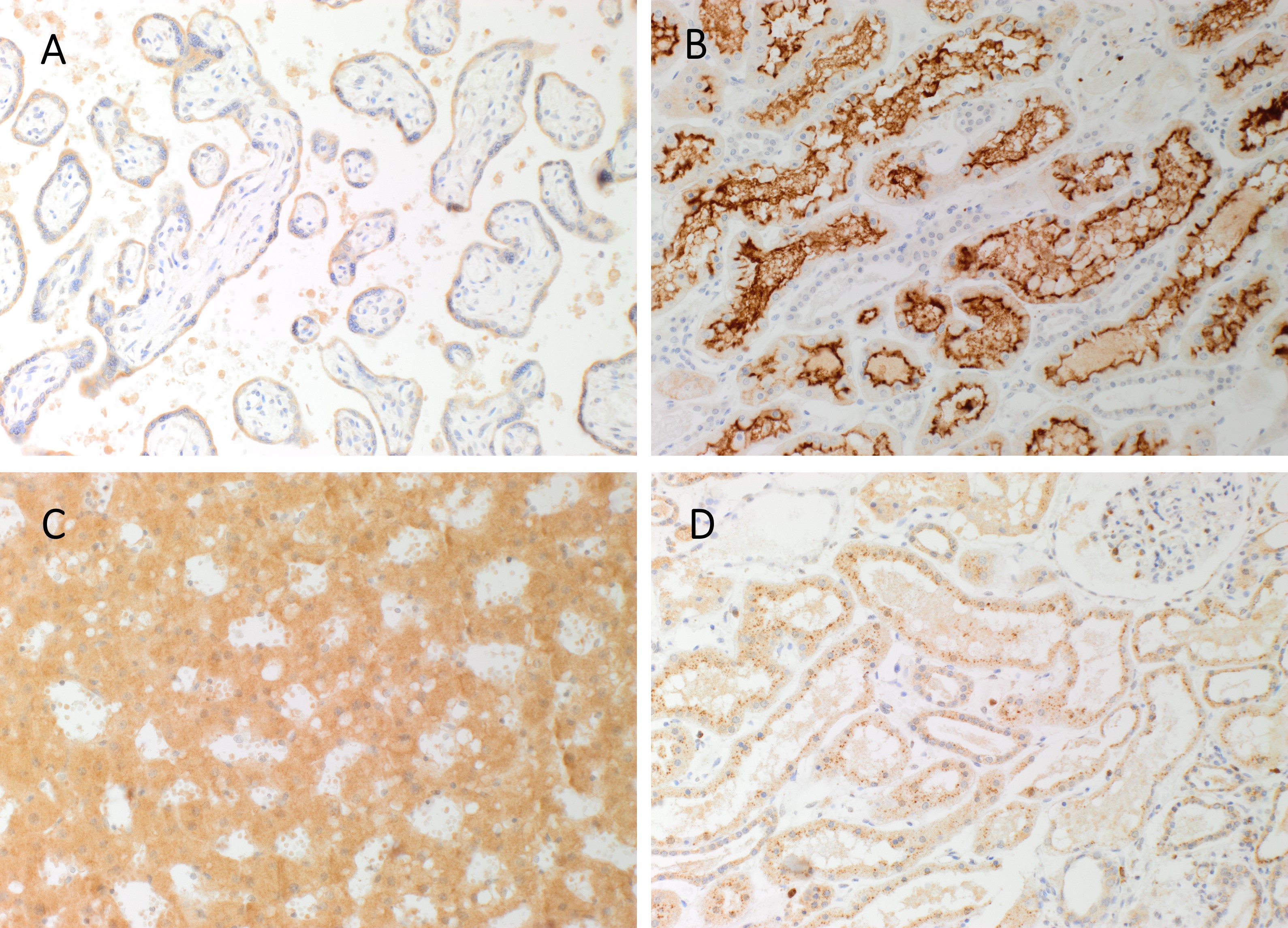

Supplement: Figure S1 — Representative 3,3-diaminobenzidine immunohistochemical-stained sections of positive control human tissues showing positive staining in placenta for PRR (A), kidney for ACE (B, brown) and ATIIR2 (D, brown), and liver for ATIIR1 (C, brown). [file image_1.jpeg]

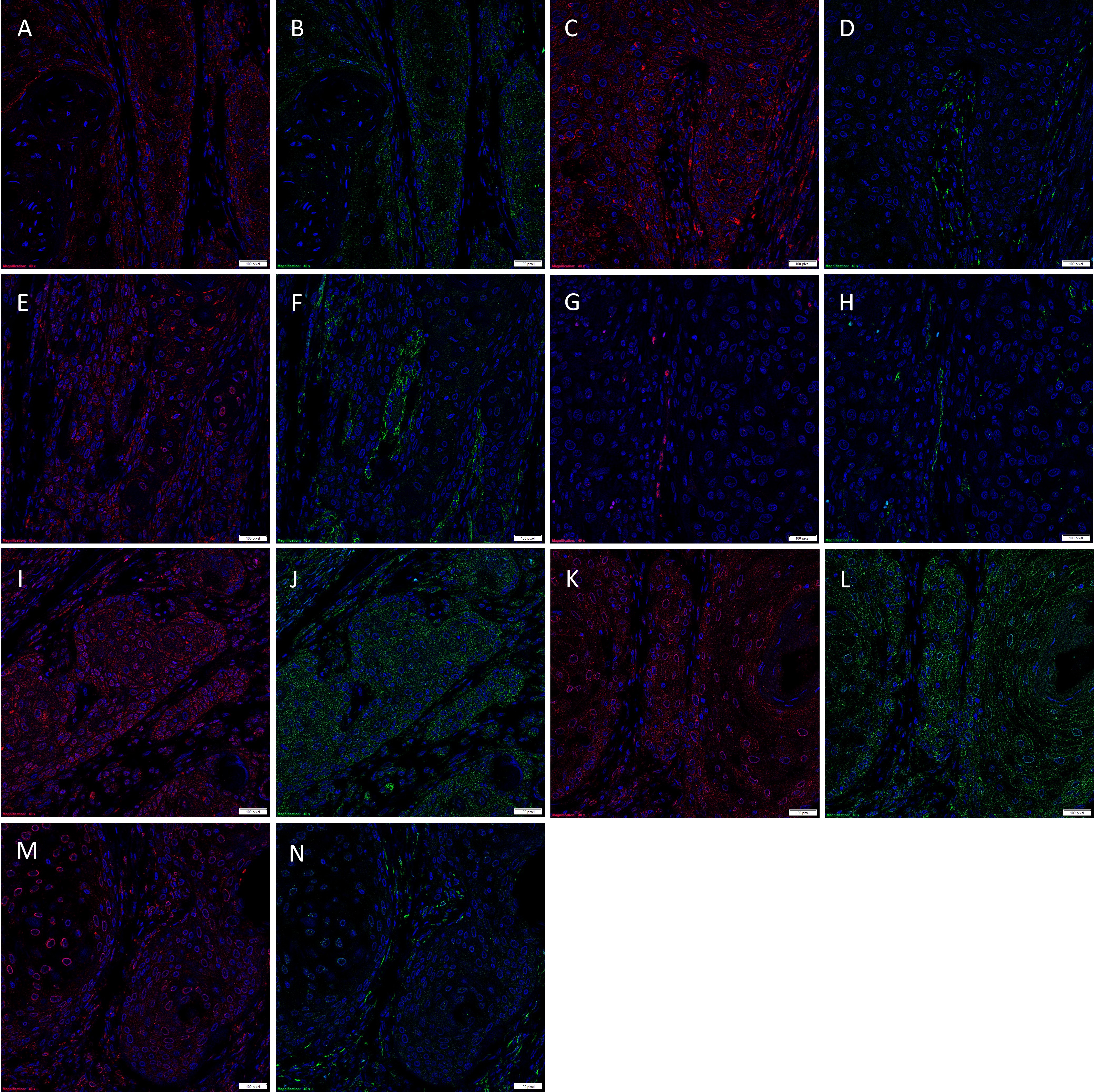

Supplement: Figure S2 — Split images of immunofluorescent immunohistochemical-stained sections moderately differentiated lip squamous cell carcinoma demonstrating the expression of for PRR (A,C, red), SALL4 (B,L, green), OCT4 (D,H,N, green), SOX2 (E,I, red), ACE (F, green), ERG (G, red), ATIIR1 (J, green), ATIIR2 (K,M, red) shown in Figure 2. Cell nuclei were counterstained with 4′,6′-diamidino-2-phenylindole (A–N, blue). Scale bars: 20 μm. [file image_2.jpeg]
